# 

**Published:** 1906-04-21

**Authors:** 


CONTENTS.

**The Uncertainties of Therapeutics** - 43
**Annotations** - 44
**Hospital Clinics—**
Diet - 45
The Heart in the Tuberculous - 46
Diseases Associated with Lymphatic Enlargement - 47
Citrate of Soda in the Feeding of Infants - 48
**Progress in Medicine and Surgery—**
Diseases of the Intestines - 48
Genito-Urinary Surgery - 50
Diseases of Children - 51
**Medical Appointments and Vacancies** - 52
**Hospital Diets in 1764** - 53
**Hospital Administration—**
A Study in Poor-law Expenditure - 54
New Poor-law Infirmary, Leicester - 55
The Problem of Radiant Heat v. Warmed Air - 58
Hospital Meetings - 59
**Editor's Letter-Box** - 59
**Notes and News** - 60

*NURSING SECTION.*

**Notes on News from the Nursing World—** Resignation of the Matron of Grimsby Hospital—Scarcity of Sisters—Nursing Institutions and Registration—Maternity Nurses at a Discount—Waitresses attired as Nurses—Changes at the Nurses Hostel—Garments and Linen—Nurses for Nice—West of England Guardians and District Nursing—South London District Nursing Association—Bolingbroke Hospital—Lectures to Midwives—The Holiday Question at Bristol—District Nursing in Cornwall—A Question of Capacity—Queen's Nurses and Small Fees—A Heavy Debt at Cardiff - 43-46
**The Nursing Outlook** - 47
**The Care and Nursing of the Insane** - 48
**The Nurses' Clinic** - 49
**Incidents in a Nurse's Life** - 50
**The Effect of Training on Character** - 51
**Central Midwives Board** - 52
**Appointments** - 52
**Everybody's Opinion** - 53
**His First Easter Dawn** - 54
**Presentations** - 54
**Death in Our Ranks** - 54
**Wants and Workers** - 54
**A Book and Its Story** - 55
**Notes and Queries** - 56
**For Reading to the Sick** - 56

